# Real-world efficacy of fimasartan vs. other angiotensin receptor blockers in combination with calcium channel blockers: a nationwide cohort study

**DOI:** 10.1186/s40885-024-00287-4

**Published:** 2024-10-01

**Authors:** Huijin Lee, Chan Soon Park, Bongseong Kim, Tae-Min Rhee, Heesun Lee, Yong-Jin Kim, Kyungdo Han, Hyung-Kwan Kim

**Affiliations:** 1 Department of Critical Care Medicine, Seoul National University Hospital, Seoul, Republic of Korea; 2 Division of Cardiology, Department of Internal Medicine, Seoul National University Hospital, Seoul, Republic of Korea; 3 Department of Statistics and Actuarial Science, Soongsil University, Seoul, Republic of Korea; 4 Division of Cardiology, Department of Internal Medicine, Seoul National University Hospital Healthcare System Gangnam Center, 152, Teheran-ro, Gangnam-gu, Seoul, Republic of Korea; 5 Department of Internal Medicine, Seoul National University College of Medicine, Seoul, Republic of Korea

**Keywords:** Hypertension, Angiotensin II inhibitor, Fimasartan, Calcium channel blocker

## Abstract

**Background:**

The antihypertensive efficacy of fimasartan was assessed based on the transition rate from a combination of calcium channel blockers (CCB) and angiotensin receptor blockers (ARB) to three-drug combination therapy, as compared to other ARBs.

**Methods:**

This nationwide cohort study used data obtained from the Korean National Health Insurance Service database. Patients who had received national health checkups within 2 years prior to January 1, 2017, and were concurrently prescribed ARBs and CCBs for > 30 days during the 6 months from January 1, 2017, to June 30, 2017 were included in the study. Patients were categorized into the ‘fimasartan group’ (those prescribed fimasartan) and the ‘non-fimasartan group’ (those prescribed ARBs other than fimasartan). The index date was set as the last day of a 30-day prescription period for ARBs and CCBs, with a subsequent 2.5-year follow-up to observe the potential addition of a third drug, such as beta-blockers or diuretics.

**Results:**

The study included 34,422 patients with a mean age of 60.3 years and 58.3% being male. The fimasartan group constituted 2.7% (*n* = 928) of the total, and the non-fimasartan group, 97.3% (*n* = 33,494). During the follow-up period, 38 patients in the fimasartan group (14.3 per 1,000 person-years) and 3,557 patients in the non-fimasartan group (42.8 per 1,000 person-years) required additional antihypertensive medications. After multivariate adjustment for age, sex, diabetes mellitus, dyslipidemia, cancer, heart failure, systolic blood pressure, and diastolic blood pressure, the fimasartan group showed a significantly lower rate of adding a third medication (hazard ratio 2.68, 95% confidence interval 1.95–3.69) compared to that of the non-fimasartan group.

**Conclusions:**

Fimasartan is associated with a lower need for additional antihypertensive drugs compared to other ARBs. This implies its greater effectiveness in hypertension management, potentially enhancing cardiovascular outcomes, and minimizing polypharmacy.

**Supplementary Information:**

The online version contains supplementary material available at 10.1186/s40885-024-00287-4.

## Background

Hypertension has a substantial prevalence affecting approximately 1 billion patients worldwide and the prevalence is increasing [1, 2]. More importantly, hypertension is a well-known risk factor for cardiovascular events [3]. Effective treatment of hypertension significantly reduces cardiovascular disease (CVD)-related events, including a decreased risk of stroke (by 35–40%), myocardial infarction (by 15–25%), and congestive heart failure (by as much as 64%) [4–6].

Despite the availability of more than 100 different medications across various pharmacological categories and large financial investments in hypertension treatment, global blood pressure (BP) control rates remain suboptimal, even in developed countries [7]. Among various factors suggested as reasons for unsatisfactory hypertension control, an expanded medication regimen has been reported to be a significant factor associated with non-compliance [8, 9]. This non-compliance could, in turn, lead to poor BP control.

Angiotensin receptor blockers (ARBs) are renowned for their efficacy and excellent tolerability [10]. Based on these qualities, ARBs stand as the preferred initial therapeutic choice for hypertension management [11–13]. Fimasartan, created by substituting the imidazole ring in losartan with a pyrimidine ring, is one of the most recent additions to the global ARB armamentarium. It has several strengths compared with other ARBs. First, it shows superior angiotensin II receptor type 1 (AT_1_) -selective binding when compared to other ARBs [14]. Second, it has an antihypertensive effect over 24 h due to its extended half-life, ranging from 10 to 18 h, ranking it among the most prolonged half-lives within the ARB class [15, 16]. A recent report showed that fimasartan has a more pronounced BP-lowering effect relative to other ARBs [17, 18]. 

In real-world clinical practice, approximately 60% of patients with hypertension are prescribed two or more antihypertensive medications. Among those, about half use combinations of ARBs and calcium channel blockers (CCBs) [19]. Considering the distinct BP-lowering effects of ARBs, it is reasonable to hypothesize that transition rates from two-drug to three-drug combination therapy may vary across ARBs; fimasartan might have a lower transition rate owing to its high potency in controlling BP.

Based on this background, we aimed to explore the efficacy of fimasartan and other ARBs in patients with hypertension who are already undergoing treatment with CCBs and ARBs by analyzing the transition rates to a three-drug combination therapy.

## Participants and methods

### Study design and study population

This nationwide cohort study used data from the Korean NHIS database. Detailed information regarding this database has been published earlier [20–22]. In essence, the NHIS is a single public insurer that covers the entire Korean population and encourages eligible Korean adults to receive general health checkups on a regular basis. Demographic information, general health checkup results, and medical history coded according to the International Classification of Diseases, Tenth Revision, Clinical Modification (ICD-10-CM) were collected and recorded in the NHIS database.

**Fig. 1:**
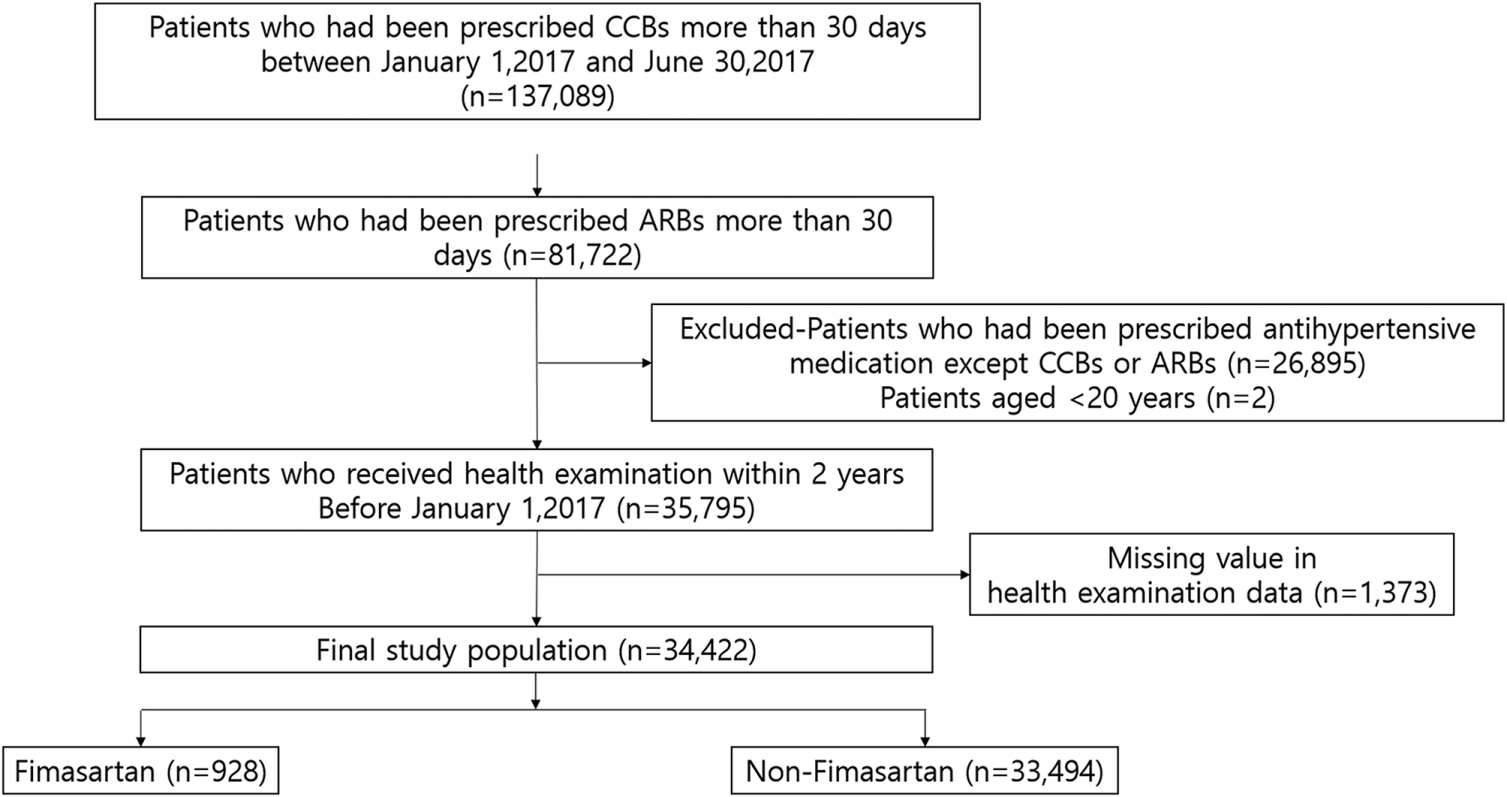
Flow chart detailing the study enrollment process. Abbreviations: CCB - calcium channel blocker; ARB - angiotensin receptor blocker

A flowchart of the study is shown in Fig. 1. Initially, we screened 137,089 patients who were prescribed CCBs for > 30 days between January 1, 2017, and June 30, 2017. Among them, 81,722 patients who were prescribed ARBs for > 30 days during the same period were identified. Patients who underwent a health checkup within 2 years prior to receiving CCB and ARB prescriptions were included. After excluding patients taking additional antihypertensive drugs, those aged < 20 years, and those with missing health data, 34,422 patients remained. Prescription of ARBs were defined as that of Fimasartan, Candesartan, Eprosartan, Irbesartan, Losartan, Olmesartan, Telmisartan, and Valsartan.

### Covariates

Patients with hypertension were identified based on one of the following criteria: (1) at least one annual claim for an antihypertensive prescription associated with ICD-10-CM codes I10-I13 and I15, derived from insurance claims data, or (2) a recorded systolic BP of ≥ 140 mmHg and/or a diastolic BP of ≥ 90 mmHg alongside the aforementioned ICD-10-CM codes [23–25]. Patients’ demographic data, comorbidities, medications, and results from general health checkups were collected and analyzed as covariates. The comorbidity definitions are summarized in Supplemental Table 1 [23]. 

### Study outcome and follow-up

The index date was defined as the last day of the 30-day ARBs and CCBs prescription period. Patients were followed up for a period of 2.5 years from the index date. The transition to three-drug combination therapy was defined as the addition of beta-blockers or diuretics during the follow-up period. Transition rates were analyzed between patients receiving fimasartan and those receiving all other ARBs (non-fimasartan), as well as between those receiving fimasartan and each individual ARB.

### Statistical analysis

Data were presented as the mean ± standard deviation for continuous variables and as counts and percentages for categorical variables. Chi-squared or Fisher’s exact tests were used for categorical variables, as appropriate. Continuous variables were analyzed using unpaired Student’s t-test, and one-way analysis of variance was used for comparisons between more than two groups. The annualized incidence rate (IR) of transitioning to three-drug combination therapy was calculated by dividing the number of new cases by the total follow-up period, and was expressed as a rate per 1,000 person-years. For the category ‘transition to three-drug combination therapy,’ we established two definitions: Definition 1 required prescriptions of CCBs, ARBs, and either beta-blockers or diuretics, within the timeframe of July 1, 2017, to December 31, 2020. Definition 2 mirrored these criteria but additionally necessitated the consistent use of the initially prescribed ARB, which was sustained until the issuance of a third antihypertensive prescription (either a beta-blocker or diuretic). Hazard ratios (HRs) and their 95% confidence intervals (CIs) were calculated using Cox proportional hazard regression models. The multivariate models were adjusted for a variety of covariates, including age, sex, systolic and diastolic BP, and comorbidities such as diabetes mellitus, dyslipidemia, atrial fibrillation, and cancer. The HRs were reported using the fimasartan group as a reference. Considering the time required to initiate antihypertensive medication, assess its efficacy, and introduce additional medications, we conducted a sensitivity analysis incorporating a 6-month lag period. To exclude biases from other cardiovascular events, we further analyzed the differences in the incidence of myocardial infarction (MI), heart failure (HF), and atrial fibrillation (AF) during follow-up between the fimasartan group and the non-fimasartan group. Additionally, we analyzed the transition rate to three-drug combination therapy in patients without a prior medical history of AF, MI, HF, and chronic kidney disease (CKD) as sensitivity analyses. Statistical significance was defined as a two-sided P-value < 0.05. All the statistical analyses were performed using SAS version 9.4 (SAS Institute, Cary, NC, USA).

## Results

### Baseline characteristics

We analyzed 34,422 patients with a mean age of 60.3 ± 11.5 years, and 20,071 patients (58.3%) were men. Among the total study population, 928 patients (2.7%) were classified into the fimasartan group and 33,494 patients (97.3%) were classified into the non-fimasartan group. The non-fimasartan group comprised patients who were prescribed candesartan, esprosartan, irbesartan, losartan, olmesartan, telmisartan, and valsartan (*n* = 1,541, 92, 590, 7,390, 5,293, 7,319, and 11,269, respectively). Baseline characteristics according to ARBs use are presented in Table 1. There were no significant differences in age, sex, or body mass index between the fimasartan and non-fimasartan groups. Regarding comorbid conditions, the fimasartan group showed unfavorable baseline characteristics, including dyslipidemia, atrial fibrillation, stroke, cancer, and heart failure, compared to the non-fimasartan group. Additionally, significant differences were observed in the systolic BP and diastolic BP between the two groups.

**Table 1:** Baseline characteristics

	Total (*n* = 34,422)	Fimasartan group (*n* = 928)	Non-Fimasartan group (*n* = 33,494)	*P*- value	Candesartan (*n* = 1,541)	Eporsartan (*n* = 92)	Irbesartan (*n* = 590)	Losartan (*n* = 7,390)	Olmesartan (*n* = 5,293)	Telmesartan (*n* = 7,319)	Valsartan (*n* = 11,269)	*P*- value
Age (years)	60.33 ± 11.5	59.7 ± 11.8	60.4 ± 11.5	0.080	60.6 ± 11.5	64.9 ± 10.4	63.2 ± 11.0	62.4 ± 11.2	59.8 ± 11.5	59.1 ± 11.3	59.9 ± 11.6	< 0.001
Sex, male	20,071 (58.3)	534 (57.5)	19,537 (58.3)	0.632	887 (57.6)	46 (50.0)	330 (55.9)	4019 (54.4)	3172 (59.9)	4486 (61.3)	6597 (58.5)	< 0.001
***Comorbidities***												
Diabetes mellitus	8203 (23.8)	213 (23.0)	7990 (23.9)	0.525	400 (26.0)	23 (25.0)	178 (30.2)	1662 (22.5)	1295 (24.5)	1645 (22.5)	2787 (24.7)	< 0.001
Dyslipidemia	15,692 (45.6)	465 (50.1)	15,227 (45.5)	0.005	744 (48.3)	60 (65.2)	283 (48.0)	3040 (41.1)	2515 (47.5)	3341 (45.7)	5244 (46.5)	< 0.001
Atrial fibrillation	276 (0.8)	15 (1.6)	261 (0.8)	0.005	17 (1.1)	4 (4.4)	8 (1.4)	58 (0.8)	41 (0.8)	52 (0.7)	81 (0.7)	< 0.001
Heart failure	239 (0.7)	15 (1.6)	224 (0.7)	0.001	28 (1.8)	0 (0)	5 (0.9)	45 (0.6)	35 (0.7)	36 (0.5)	75 (0.7)	< 0.001
Prior MI	94 (0.3)	3 (0.3)	91 (0.3)	0.766	7 (0.5)	0 (0)	4 (0.7)	20 (0.3)	11 (0.2)	21 (0.3)	28 (0.3)	0.442
PAD	5652 (16.4)	140 (15.1)	5512 (16.5)	0.266	262 (17.0)	13 (14.1)	91 (15.4)	1311 (17.7)	823 (15.6)	1063 (14.5)	1949 (17.3)	< 0.001
Stroke	1541 (4.5)	62 (6.7)	1479 (4.4)	0.001	71 (4.6)	14 (15.2)	49 (8.3)	299 (4.1)	332 (6.3)	287 (3.9)	427 (3.8)	< 0.001
Cancer	1074 (3.1)	43 (4.6)	1031 (3.1)	0.007	54 (3.5)	2 (2.2)	17 (2.9)	270 (3.7)	155 (2.9)	196 (2.7)	337 (3.0)	0.004
***Health exam parameters***												
BMI (kg/m2)	25.6 ± 3.4	25.6 ± 3.5	25.6 ± 3.4	0.563	25.6 ± 3.5	25.3 ± 3.7	25.3 ± 3.2	25.3 ± 3.39	25.8 ± 3.4	25.7 ± 3.4	25.7 ± 3.4	< 0.001
Waist circumference (cm)	86.3 ± 8.8	86.2 ± 8.9	86.3 ± 8.8	0.882	86.2 ± 9.1	85.3 ± 8.8	86.1 ± 8.8	85.7 ± 8.7	86.6 ± 8.7	86.5 ± 8.8	86.3 ± 8.8	< 0.001
Systolic BP (mmHg)	132.6 ± 15.5	133.8 ± 16.2	132.6 ± 15.5	0.016	133.7 ± 15.6	131.9 ± 16.4	132.3 ± 14.2	132.1 ± 15.1	131.9 ± 15.8	132.4 ± 15.9	133.1 ± 15.5	< 0.001
Diastolic BP (mmHg)	81.0 ± 10.7	82.0 ± 11.5	81.0 ± 10.7	0.004	81.7 ± 11.2	79.5 ± 10.1	80.5 ± 10.1	80.3 ± 10.2	80.6 ± 10.9	81.1 ± 10.9	81.4 ± 10.6	< 0.001
Fasting glucose (mg/dL)	110.0 ± 31.0	109.4 ± 34.3	110.1 ± 30.9	0.485	109.9 ± 31.7	108.0 ± 26.6	111.1 ± 28.7	108.8 ± 29.1	110.7 ± 30.9	109.8 ± 30.8	110.7 ± 32.2	0.004
Total cholesterol (mg/dL)	190.8 ± 39.6	192.2 ± 41.7	190.8 ± 39.6	0.285	190.3 ± 39.4	180.5 ± 36.7	186.6 ± 38.4	191.1 ± 38.4	190.5 ± 39.9	191.6 ± 39.9	190.5 ± 40.0	0.007
HDL-C (mg/dL)	53.1 ± 14.0	53.3 ± 15.1	53.1 ± 13.9	0.592	52.7 ± 13.3	54.9 ± 15.1	51.9 ± 13.2	53.3 ± 14.5	53.0 ± 13.8	52.9 ± 13.8	53.1 ± 13.8	0.157
LDL-C (mg/dL)	108.8 ± 37.1	109.4 ± 37.7	108.8 ± 37.1	0.618	108.4 ± 35.8	98.7 ± 29.7	105.6 ± 34.6	109.5 ± 36.4	108.1 ± 36.5	110.0 ± 38.3	108.1 ± 37.4	< 0.001
eGFR (ml/min/1.73m^2^)	89.0 ± 50.8	87.9 ± 44.0	89.1 ± 50.9	0.511	88.3 ± 57.4	86.1 ± 21.5	82.6 ± 27.6	86.4 ± 36.2	89.4 ± 59.5	90.6 ± 52.3	90.1 ± 54.0	< 0.001

Abbreviation: MI, myocardial infarction; PAD, peripheral artery disease; BMI, body mass index; BP, blood pressure; HDL-C, high-density lipoprotein cholesterol; LDL-C, low-density lipoprotein cholesterol; eGFR, estimated glomerular filtration rate

### Different transition rates to three-drug combination therapy

During a median follow-up of 2.1 years (interquartile range 2.05–2.35 years), 3,595 patients (10.5%) transitioned to three-drug combination therapy. Significant differences in the transition to three-drug combination therapy were observed between the fimasartan and non-fimasartan groups (*P* < 0.001), as well as across ARBs (*P* < 0.001) (Table 2). Notably, the patients taking fimasartan exhibited a remarkably lower rate of adding other antihypertensive medications; only 38 patients (4.1%) taking fimasartan required additional medication. In contrast, eprosartan (29.4%) and irbesartan (29.8%) exhibited the highest transition rates in three-drug combination therapy, followed by candesartan (21.5%) and losartan (20.4%). Olmesartan (5.8%), telmisartan (6.3%), and valsartan (6.6%) showed comparatively lower rates.

**Table 2:** Time to addition of third antihypertensive medication: comparison among angiotensin II receptor blockers

	Fimasartan (*n* = 928)	Candesartan (*n* = 1,541)	Eprosartan (*n* = 92)	Irbesartan (*n* = 590)	Losartan (*n* = 7,390)	Olmesartan (*n* = 5,293)	Telmisartan (*n* = 7,319)	Valsartan (*n* = 11,269)	*P*-value
**Third medication addition rate,** *n*** (%)**									
definition 1	58 (6.25)	372 (24.14)	29 (31.52)	186 (31.53)	1609 (21.77)	441 (8.33)	589 (8.05)	1049 (9.31)	< 0.001
definition 2	38 (4.09)	332 (21.54)	27 (29.35)	176 (29.83)	1507 (20.39)	308 (5.82)	462 (6.31)	745 (6.61)	< 0.001
**Time to third medication addition**,** days**									
definition 1 (Median [IQR1 - IQR3])	243 (194, 547)	203 (187, 239.5)	210 (188, 280)	194 (184, 218)	201 (186, 241)	225 (188, 552)	238 (193, 538)	238 (192, 548)	< 0.001
definition 2 (Median [IQR1 - IQR3])	213.5 (184, 278)	198 (186, 218)	203 (187, 240)	193 (183, 211)	198 (186, 231)	198 (185, 242.5)	213 (190, 357)	204 (188, 267)	< 0.001

Definition 1: A claim exists for the prescription of CCBs, ARBs, and beta-blockers or diuretics between July 1, 2017, and December 31, 2020

Definition 2: A claim exists for the prescription of CCBs, ARBs, and beta-blockers or diuretics between July 1, 2017, and December 31, 2020, with a consistent ARB since index time, maintained until the third drug claim

Abbreviation: SD, standard deviation; IQR, interquartile

The time taken to use additional antihypertensive medications was also significantly different across ARBs (Table 2). Indeed, patients taking fimasartan showed a longer duration of two-drug combination therapy before additional medication was required, with a median time of 213.5 days (interquartile range, 184–278 days), indicating a longer interval than that for most other ARBs. Irbesartan demonstrated the shortest duration (median: 193 days; interquartile range, 183–211 days).

**Fig. 2:**
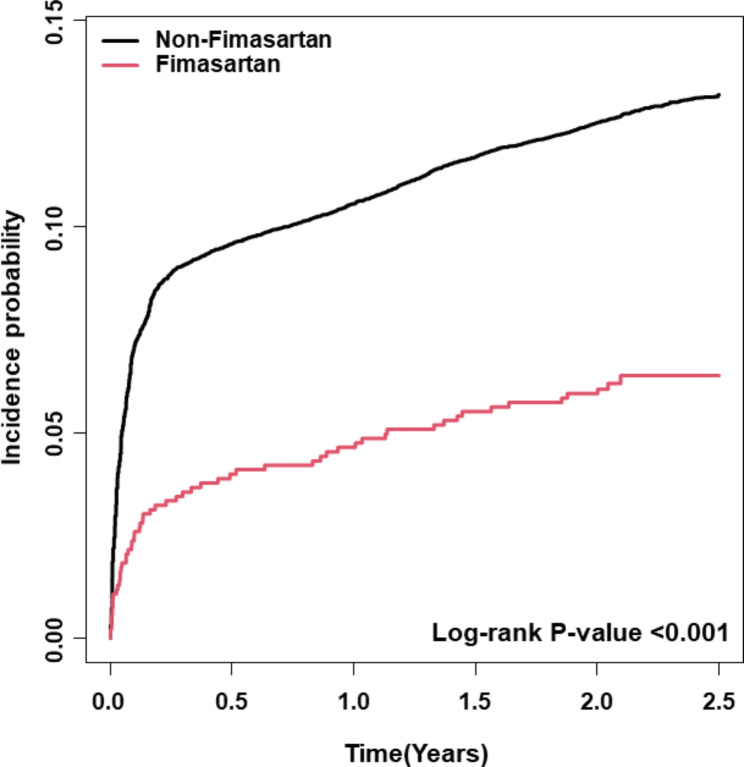
Survival analysis contrasting the transition rate to a three-drug combination therapy regimen between the fimasartan and non-fimasartan cohorts

**Fig. 3:**
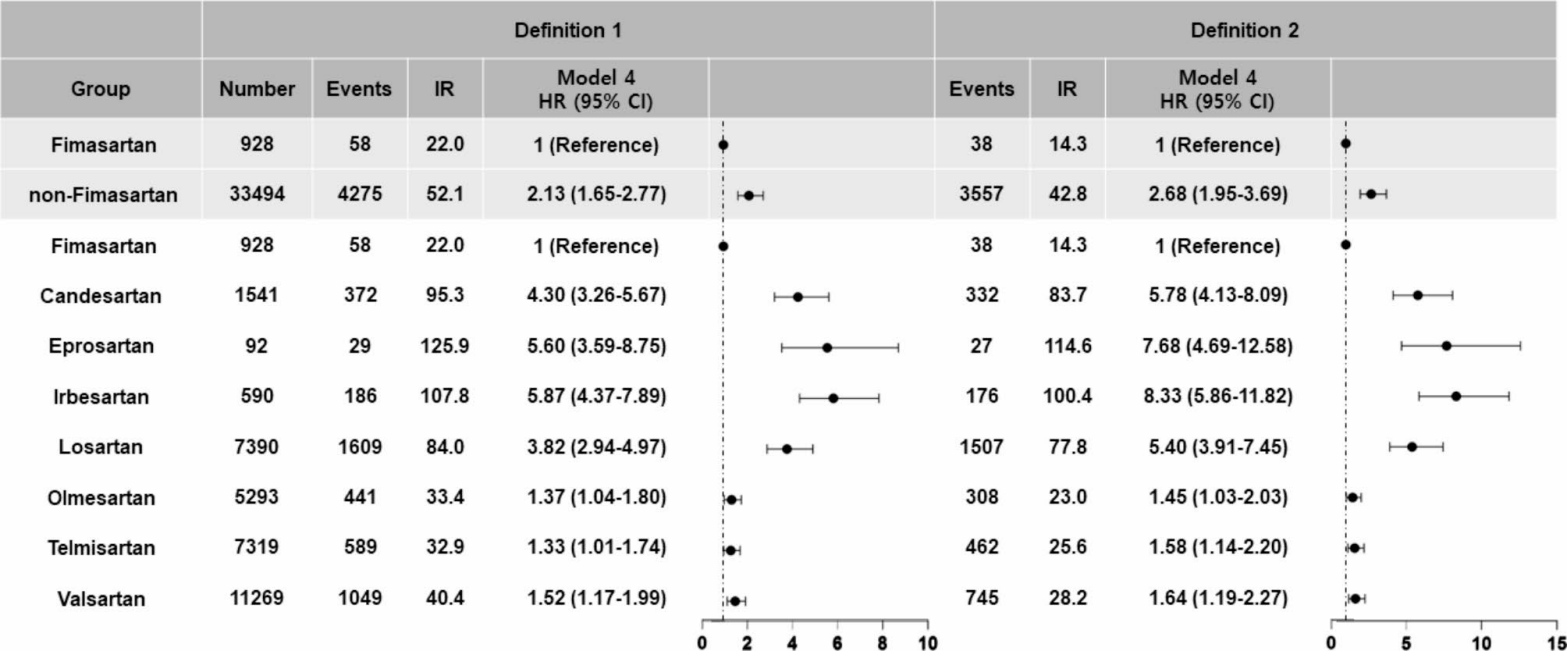
Multivariate Cox-proportional hazard regression analysis assessing the transition rate to three-drug combination therapy across fimasartan and non-fimasartan groups, and the aggregate of all angiotensin receptor blockers. The fimasartan group is utilized as a reference. Incidence rate is expressed per 1,000 person-years. Abbreviations: CI = confidence interval; HR = hazard ratio; IR = incidence rate

The annualized IRs of each ARB were quite different. The fimasartan group showed an IRs of 14.26 per 1,000 PY, whereas the non-fimasartan group showed an IRs of 42.76 per 1,000 PY. As shown in the Kaplan-Meier curves (Fig. 2), the probability of taking additional antihypertensive medications was significantly lower in the fimasartan group than in the non-fimasartan group (log-rank *P* < 0.001). After multivariable adjustment, the non-fimasartan group consistently showed significantly higher transition rates to three-drug combination therapy (HR 2.68, 95% CI 1.95–3.69) compared to the fimasartan group (Fig. 3). These results highlight the potential clinical efficacy of fimasartan in minimizing the need for additional antihypertensive agents. When comparing the rate of transition in the three-drug combinations involving fimasartan and telmisartan (Supplemental Fig. 1A, *p* = 0.047) and fimasartan and olmesartan (Supplemental Fig. 1B, *p* = 0.027), the rate of transition in the three-drug combination was statistically significantly lower for fimasartan than for both telmisartan and olmesartan. The results of sensitivity analysis with the 6-month landmark analysis were consistent with the main results (Supplemental Fig. 2).

We further compared the transition rate to three-drug combination therapy for each ARB, with patients taking fimasartan as the reference group. Supplemental Fig. 3 illustrates the trend of adding antihypertensive medications to each ARB. In multivariate analysis, all other ARBs showed significantly higher risks of additional antihypertensive medications than that of fimasartan. Specifically, candesartan (HR 5.78, 95% CI 4.13–8.09), eprosartan (HR 7.68, 95% CI 4.69–12.58), and irbesartan (HR 8.33, 95% CI 5.86–11.82) demonstrated remarkably higher transition rates to three-drug combination therapy compared to fimasartan. Regarding olmesartan, telmisartan, and valsartan, the relative magnitude of increased risks for adding additional antihypertensive medications was comparatively small compared to that of candesartan, eprosartan and irbesartan, but these also showed significantly higher risks compared to fimasartan (HR 1.45, 95 CI 1.03–2.03; HR 1.58, 95% CI 1.14–2.20; and HR 1.64, 95% CI 1.19–2.27, respectively) (Fig. 3). This result was consistently observed in the sensitivity analysis with a 6-month lag period. (Supplemental Fig. 3) and in the analysis excluding patients with a past medical history of MI, HF, AF, and CKD (Supplemental Tables 2 and Supplemental Fig. 4).

To exclude the biases from the newly developed cardiovascular events except hypertension, we further analyzed the differences in the incidence of MI, HF, and AF during follow-up between the two groups. Supplemental Fig. 5 illustrates the multivariate analysis of these four outcomes, showing similar risks in the non-fimasartan group for MI (HR: 0.79, 95 CI: 0.35–1.79), HF (HR: 0.74, 95 CI: 0.44–1.24), AF (HR: 0.87, 95 CI: 0.48–1.59), and the composite outcome (HR: 0.77, 95 CI: 0.53–1.13).

## Discussion

We evaluated the efficacy of ARBs by examining the rate at which patients transitioned from a combination of CCBs and ARBs to a triple-drug regimen. The key findings are as follows: First, fimasartan was associated with a lower frequency of introducing a third antihypertensive agent compared to other ARBs. Second, patients on fimasartan had a longer median duration (778 days) before the addition of a third antihypertensive agent than those on other ARBs (median 764 days). This trend persisted even after adjusting for covariates, with fimasartan showing a decreased likelihood of requiring a third antihypertensive medication. It is important to note that despite unfavorable baseline characteristics in the fimasartan group, there was less need for an additional antihypertensive agent. These results suggest that fimasartan may offer superior antihypertensive efficacy, as evidenced by both a lower incidence of, and delayed requirement for, additional antihypertensive medication.

Because of the unacceptably high global disease burden of hypertension, numerous efforts have been made to improve clinical outcomes, demanding lifelong compliance with treatment [26, 27]. However, most patients with hypertension remain asymptomatic despite the increased risk of cardiovascular events. Consequently, adherence to antihypertensive medications, which may not provide immediate benefits, is crucial for these patients. The documented correlation between complex medication schedules, involving multiple drugs and daily doses, and reduced compliance underscores the challenge [28–30]. This complexity is further compounded when considering comorbidities, leading to intricate drug regimens involving multiple medications and doses [31]. In consideration of this, it is essential for physicians to simplify the antihypertensive medication regimen while preserving equivalent medical and antihypertensive benefits to enhance outcomes.

ARBs have a shared molecular structure that contributes to their class effect [32], yet variations exist, leading to different clinical benefits. The number of hydrogen bonds, which affect binding affinity, varies among ARBs and influences their antihypertensive effects [33]. Fimasartan is distinct in the replacement of losartan’s imidazole ring with a pyrimidine ring, resulting in enhanced AT_1_-selective binding compared to other ARBs [14]. It also has an extended half-life and potent BP-lowering effect [15, 16, 18]. Considering that fimasartan belongs to the later generation of ARBs, comparisons are made with with earlier ARBs to establish its antihypertensive efficacy. The antihypertensive efficacy of fimasartan has been investigated compared to losartan [34], valsartan [16, 35, 36], and candesartan [37] and it is clear that fimasartan has comparable or superior BP-lowering effects. A previous head-to-head study clearly demonstrated that fimasartan has superior efficacy in BP reduction compared with valsartan [38]. As expected from its novel molecular characterization, fimasartan has consistently demonstrated a more pronounced BP-lowering effect than other ARBs in clinical studies [15, 16, 18]. 

The use of the conversion rate to the three-drug combination therapy as a surrogate measure in our study offers a novel approach for evaluating the effectiveness of antihypertensive medications, bypassing the traditional method of direct BP measurement. Additionally, the potential of this conversion rate as an indicator of patient compliance with antihypertensive medications is noteworthy. Our cohort of > 30,000 participants is a strength, although the implications and broader applications of these findings warrant further exploration.

Our study had several limitations. First, as this was an observational study, unmeasured confounders may have influenced our results. Second, the study, which was based on the NHIS database, encountered limitations in accessing data on the degree of BP change from the index date to the prescription of a third antihypertensive medication. However, it is important to note that our findings align with a previous study that reported the effectiveness of fimasartan in lowering BP compared to other ARBs. Additionally, this study was conducted only in South Korea, and thus, generalizing the findings to global populations with diverse genetic and environmental backgrounds should be approached with caution. Another limitation is the absence of data regarding the discontinuation of ARB prescriptions. While we included individuals who were prescribed ARBs for 30 days or more within the inclusion period, we were unable to ascertain the subsequent discontinuation status of these prescriptions. In the future, research on more specific topics, such as cost-effectiveness, will be necessary.

## Conclusions

The use of fimasartan is associated with a significantly lower transition rate to three-drug combination therapy and extends the time to the incorporation of a third drug into the treatment regimen. The antihypertensive efficacy of fimasartan in the management of hypertension minimizes the need for additional medications.

## Electronic supplementary material

Below is the link to the electronic supplementary material.

Supplementary Material 1

## Data Availability

All raw data were accessible from designated terminals approved by the National Health Insurance Service (NHIS). On reasonable request from the corresponding author, the data will be available under approval and oversight by the NHIS.

## Abbreviations

CVD: Cardiovascular disease
BP: Blood pressure
ARBs: Angiotensin receptor blockers
AT1: Angiotensin II receptor type 1
CCBs: Calcium channel blockers
NHIS: National Health Insurance Service
ICD-10-CM: International Classification of Diseases, Tenth Revision, Clinical Modification
IR: Incidence rate
HRs: Hazard ratios
CIs: Confidence intervals
