# Supplementary material for: Real-world efficacy of fimasartan vs. other angiotensin receptor blockers in combination with calcium channel blockers: a nationwide cohort study
Source: Clin Hypertens. 2024 Oct 1;30:28. doi: 10.1186/s40885-024-00287-4 (PMC11443630; doi:10.1186/s40885-024-00287-4)
Supplement: Supplementary file 1 — Supplementary Material 1 [file 40885_2024_287_MOESM1_ESM.docx]

**Supplementary Online Content**

**Supplemental Table 1.** Definitions of Covariates

**Supplemental Table 2.** Time to Addition of Third Antihypertensive Medication: Comparative Analysis of Angiotensin II Receptor Blockers in Patients without Baseline AF, MI, HF, or CKD

**Supplemental Figure 1**. Kaplan-Meier Curves Comparing the Transition Rate to Three-Drug Therapy for Each Angiotensin Receptor Blocker: Fimasartan, Telmisartan, and Olmesartan

Supplemental Figure 2. Sensitivity Analysis with 6-Month Landmark Analysis: Multivariable Hazard Regression Analysis to Assess the Rate of Adding a Third Drug at the Landmark Time: Fimasartan vs. Non-Fimasartan vs. All ARBs

**Supplemental Figure 3**. Kaplan-Meier Curves Comparing the Transition Rate to Three-Drug Therapy for Each Angiotensin Receptor Blockers

**Supplemental Figure 4**. Incidence of 3-Drug Transition in a Population Excluding Patients with a History of Atrial Fibrillation, Myocardial Infarction, Heart Failure, or Chronic Kidney Disease: Cox Regression Analysis

**Supplemental Figure 5**. Incidence of Cardiovascular Events in Fimasartan vs. Non-Fimasartan Groups: Cox Regression Analysis Among Patients Without Prior MI, HF, or AF

**Supplemental Table 1.** Definitions of Covariates

| **Diagnosis** | ***ICD-10-CM* code and definition** | **Diagnostic definition** |
| --- | --- | --- |
| **Hypertension and Comorbidities** |  |  |
| **Hypertension** | I10-I13, I15; and minimum 1 prescription of anti-hypertensive drug (thiazide, loop diuretics, aldosterone antagonist, alpha-/beta-blocker, calcium-channel blocker, angiotensin-converting enzyme inhibitor, angiotensin II receptor blocker). | Admission≥1 or outpatient department≥2 |
|  | or systolic/diastolic blood pressure ≥ 140/90 mmHg | Based on the results of 2^nd^ health exam |
| **Diabetes mellitus** | E11-E14; and minimum 1 prescription of anti-diabetic drugs (sulfonylureas, metformin, meglitinides, thiazolidinediones, dipeptidyl peptidase-4 inhibitors, α-glucosidase inhibitors, and insulin). | Admission≥1 or outpatient department≥2 |
|  | or fasting glucose level ≥ 126 mg/dL | Based on the results of 2^nd^ health exam |
| **Dyslipidemia** | E78 | Admission or outpatient department≥1 |
|  | or Total cholesterol ≥ 240 mg/dL | Based on the results of 2^nd^ health exam |
| **Atrial fibrillation** | I48.0-48.4, I48.9 | Admission≥1 or outpatient department≥2 |
| **Heart failure** | I50 | Admission or outpatient department≥1 |
| **Vascular disease** |  |  |
| **Prior MI** | I21, I22 | Admission or outpatient department≥1 |
| **PAD** | I70, I73 | Admission or outpatient department≥2 |
| **Ischemic stroke** | I63, I64 | Admission≥1 and brain imaging (CT or MRI) ≥1 |
| **Intracranial hemorrhage** | I60-62 | Admission≥1 or RBC transfusion≥1 |
| **Cancer** | C00-97 and RID code (V193) | Admission or outpatient department≥1 |

# Abbreviation: ICD-10-CM, International Classification of Diseases, Tenth Revision, Clinical Modification; MI, myocardial infarction; PAD, peripheral artery disease; CT, computed tomography; MRI, magnetic resonance image; RBC, red blood cell; RID, rare and intractable disease.

**Supplemental Table 2.** Time to Addition of Third Antihypertensive Medication: Comparative Analysis of Angiotensin II Receptor Blockers in Patients without Baseline AF, MI, HF, or CKD

|  | **Fimasartan**  **(n=829)** | **Candesartan**  **(n=1,342)** | **Eprosartan**  **(n=81)** | **Irbesartan**  **(n=485)** | **Losartan**  **(n=6,627)** | **Olmesartan**  **(n=4,742)** | **Telmisartan**  **(n=6,756)** | **Valsartan**  **(n=10,272)** | **P-value** |
| --- | --- | --- | --- | --- | --- | --- | --- | --- | --- |
| **Third medication addition rate, n (%)** |  |  |  |  |  |  |  |  |  |
| definition 1 | 46(5.55) | 324(24.14) | 21(25.93) | 156(32.16) | 1422(21.46) | 387(8.16) | 531(7.86) | 918(8.94) | <0.001 |
| definition 2 | 30(3.62) | 289(21.54) | 20(24.69) | 149(30.72) | 1338(20.19) | 271(5.71) | 418(6.19) | 646(6.29) | <0.001 |
| **Time to third medication addition, days** |  |  |  |  |  |  |  |  |  |
| definition 1 (Median [IQR1 - IQR3]) | 243 (194, 547) | 203 (187, 238.5) | 210 (188, 240) | 193 (183, 217) | 200(186,240) | 229(189,558) | 238(194,535) | 239(191,546) | <0.001 |
| definition 2 (Median [IQR1 - IQR3]) | 215.5(183,301) | 197(186,218) | 202.5(187.5,239) | 193(183,211) | 198(186,230) | 200(186,244) | 214(190,356) | 203(188,263) | <0.001 |

Definition 1: A claim exists for the prescription of CCBs, ARBs, and beta-blockers or diuretics between July 1, 2017, and December 31, 2020.

Definition 2: A claim exists for the prescription of CCBs, ARBs, and beta-blockers or diuretics between July 1, 2017, and December 31, 2020, with a consistent ARB since index time, maintained until the third drug claim.

Abbreviation: AF, atrial fibrillation; MI, myocardial infarction; HF, heart failure; CKD, chronic kidney disease; SD, standard deviation; IQR, interquartile.

**Supplemental Figure 1.** Kaplan-Meier Curves Comparing the Transition Rate to Three-Drug Therapy for Each Angiotensin Receptor Blocker: Fimasartan, Telmisartan, and Olmesartan

**
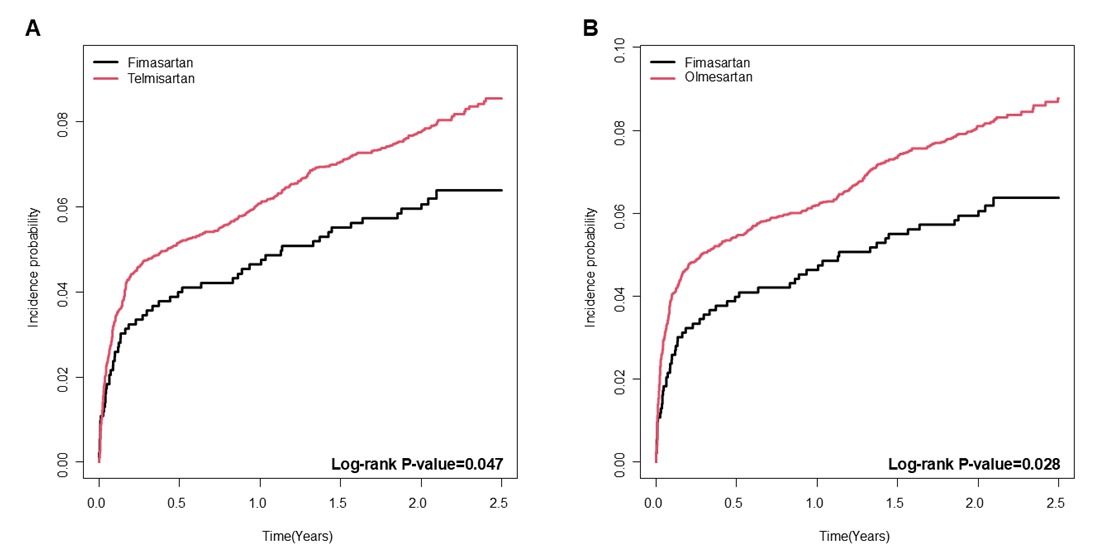
**

**Supplemental Figure 2.** Sensitivity Analysis with 6-Month Landmark Analysis: Multivariable Hazard Regression Analysis to Assess the Rate of Adding a Third Drug at the Landmark Time: Fimasartan vs. Non-Fimasartan vs. All ARBs


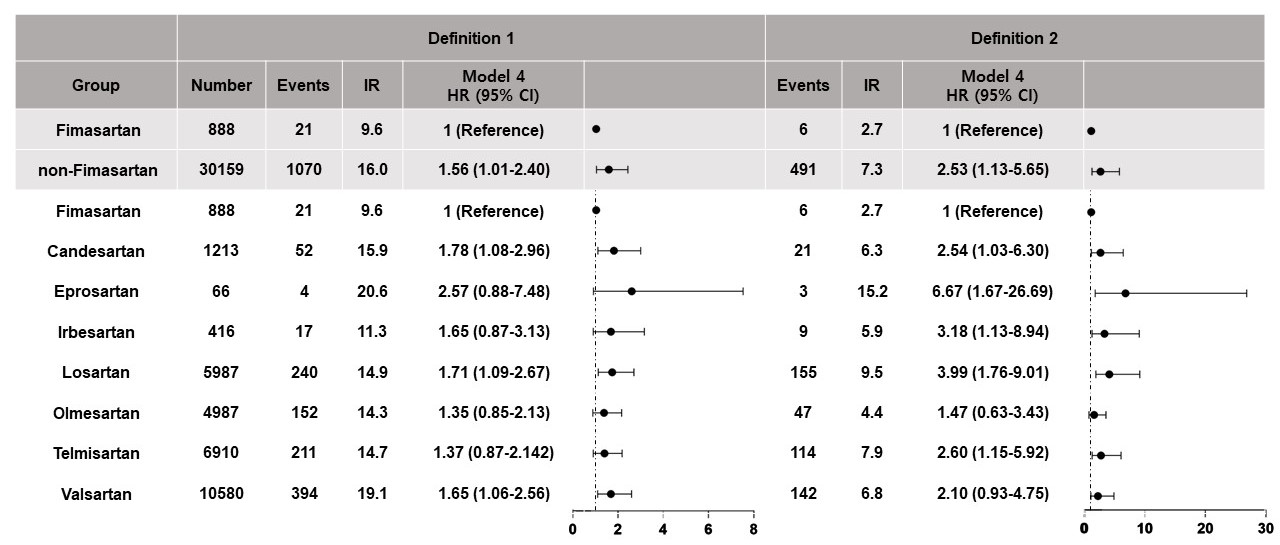


Abbreviation: IR, incidence rate; CI, confidence interval; HR, hazard ratio

**Supplemental Figure 3.** Kaplan-Meier Curves Comparing the Transition Rate to Three-Drug Therapy for Each Angiotensin Receptor Blockers


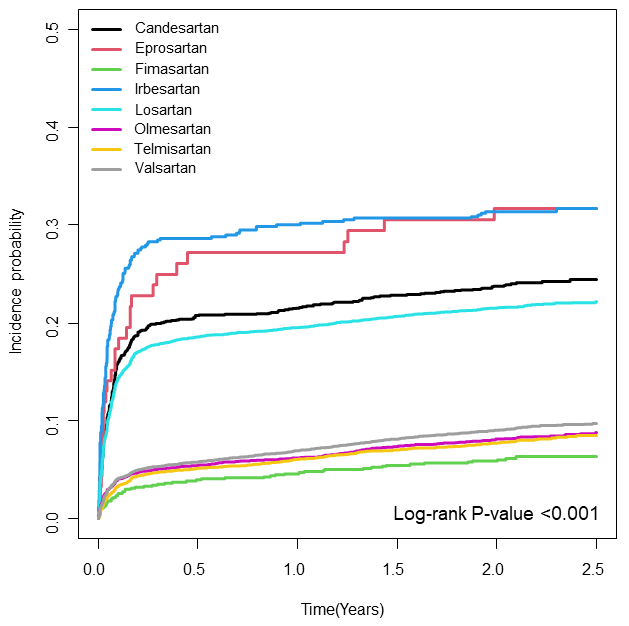


**Supplemental Figure 4.** Incidence of 3-Drug Transition in a Population Excluding Patients with a History of Atrial Fibrillation, Myocardial Infarction, Heart Failure, or Chronic Kidney Disease: Cox Regression Analysis

**
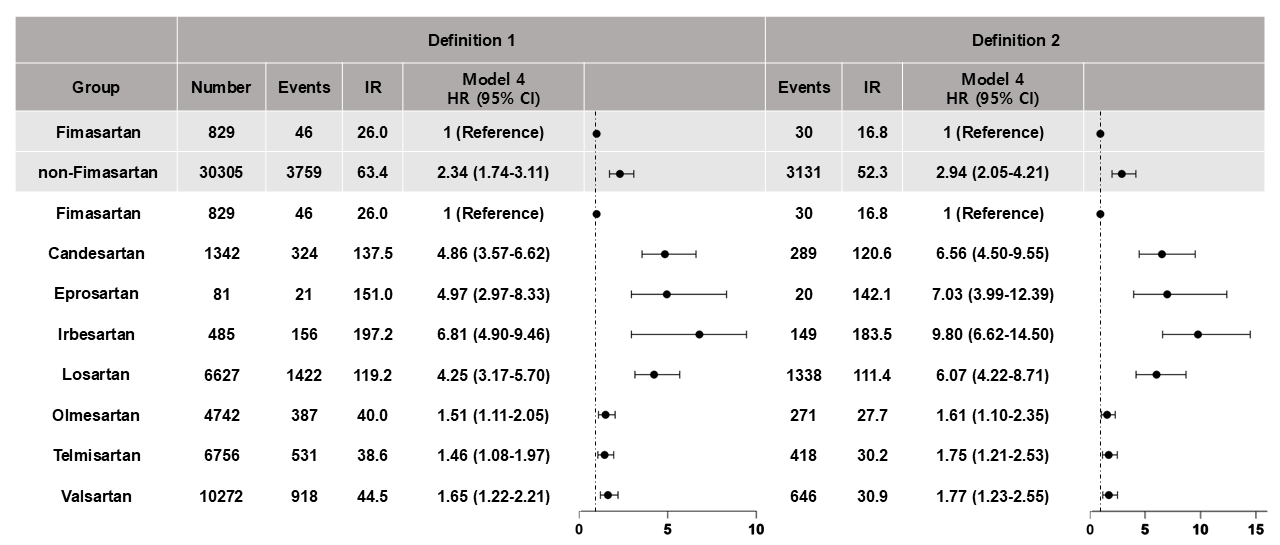
**

Abbreviation: IR, incidence rate; CI, confidence interval; HR, hazard ratio

**Supplemental Figure 5.** Incidence of Cardiovascular Events in Fimasartan vs. Non-Fimasartan Groups: Cox Regression Analysis Among Patients Without Prior MI, HF, or AF

**
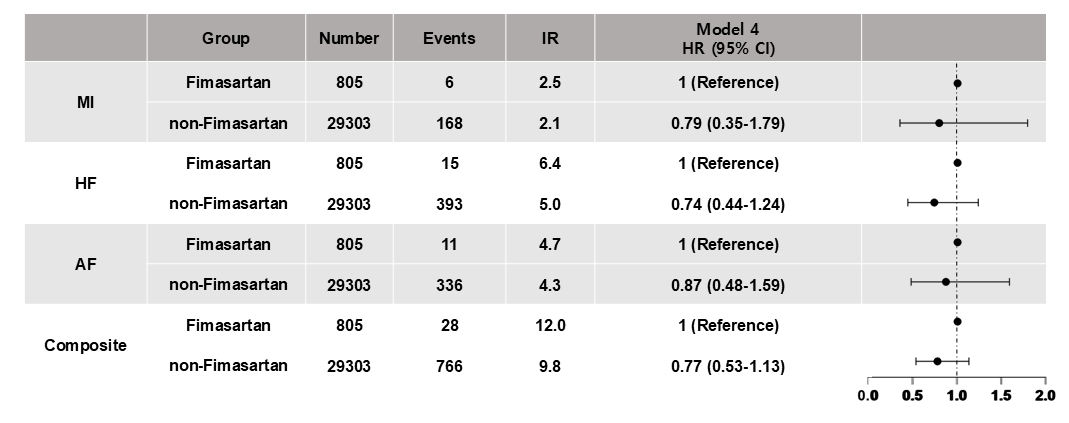
**

Abbreviation: IR, incidence rate; CI, confidence interval; HR, hazard ratio
